# The Conductor As Visual Guide: Gesture and Perception of Musical Content

**DOI:** 10.3389/fpsyg.2016.01049

**Published:** 2016-07-08

**Authors:** Anita B. Kumar, Steven J. Morrison

**Affiliations:** Laboratory for Music Cognition, Culture and Learning, School of Music, University of Washington, SeattleWA, USA

**Keywords:** conducting, audio-visual interaction, gesture, music perception, music ensembles

## Abstract

Ensemble conductors are often described as embodying the music. Researchers have determined that expressive gestures affect viewers’ perceptions of conducted ensemble performances. This effect may be due, in part, to conductor gesture delineating and amplifying specific expressive aspects of music performances. The purpose of the present study was to determine if conductor gesture affected observers’ focus of attention to contrasting aspects of ensemble performances. Audio recordings of two different music excerpts featuring two-part counterpoint (an ostinato paired with a lyric melody, and long chord tones paired with rhythmic interjections) were paired with video of two conductors. Each conductor used gesture appropriate to one or the other musical element (e.g., connected and flowing or detached and crisp) for a total of sixteen videos. Musician participants evaluated 8 of the excerpts for Articulation, Rhythm, Style, and Phrasing using four 10-point differential scales anchored by descriptive terms (e.g., *disconnected* to *connected*, and *angular* to *flowing.*) Results indicated a relationship between gesture and listeners’ evaluations of musical content. Listeners appear to be sensitive to the manner in which a conductor’s gesture delineates musical lines, particularly as an indication of overall articulation and style. This effect was observed for the lyric melody and ostinato excerpt, but not for the chords and interjections excerpt. Therefore, this effect appears to be mitigated by the congruence of gesture to preconceptions of the importance of melodic over rhythmic material, of certain instrument timbres over others, and of length between onsets of active material. These results add to a body of literature that supports the importance of the visual component in the multimodal experience of music performance.

## Introduction

Previous research has established a link between the visual and auditory perceptions of live music performance, especially with regards to the physical gestures of performers. Measured responses to a diverse array of performances by jazz and popular singers, pianists, clarinetists, percussionists playing marimba, and unconducted chamber ensembles have all demonstrated a visual dominance in perceptions of performance quality, expressivity, and emotion (Thompson et al., 2005; Thompson and Russo, 2007; Broughton and Stevens, 2009; Davidson, 2012; Thompson and Luck, 2012; Connell et al., 2013; Tsay, 2014).

Music is a multimodal phenomenon and the observation of movement or gesture is a critical component of the way it is perceived. In a meta-analysis of fifteen studies into the perception of auditory and visual components of music, Platz and Kopiez (2012, p. 75) concluded that “the visual component is not a marginal phenomenon in music perception, but an important factor in the communication of meaning”. Visual information is also critical in the adjudication of music performance, as demonstrated by Tsay (2013, p. 14580) who found in seven separate experiments that selection of winners in competitive music settings was based more on visual than auditory information: “The results highlight our natural, automatic, and non-conscious dependence on visual cues. The dominance of visual information emerges to the degree that it is over-weighted relative to auditory information, even when sound is consciously valued as the core domain content”.

In discussing the relationship between the visual and the auditory, Windsor (2011, p. 50) identified a disconnect between the perception of the observer and that of the musician, in that musicians understand the actions they take in an intimate way that is unobservable to the audience. Windsor also suggested that gestures can occur in parallel: “The combined audibility and visibility of gesture in musical performance creates a rich possibility for combining parallel gestures across or within modalities”. Graybill (2011) highlights the ways in which a personification of musical motifs as characters or agents can be tracked amongst members of a string ensemble through musical gesture related in the score, and therefore physical gesture in performance. Multiplicity of gestures and characters, and changes within each, can lead to distraction from the overarching theme and discussion in polyphonic music (Gritten, 2011). From the observer’s perspective, then, the visually perceived gestures (showing “cause” of auditory gestures, according to Windsor) may become critical to discerning what is important.

Listeners attend to a variety of musical concepts simultaneously. In an active listening activity, such as notating music that is heard, attending to rhythm first before pitch improves dictation accuracy (Beckett, 1997). Furthermore, musically untrained listeners are able to discern large-scale structures, such as musical tensions and relaxations similarly to trained listeners, as well as culturally specific melodic expectancy (Bigand, 2003; Bigand and Poulin-Charronnat, 2006). Gregory (1990) found that in listening to polyphonic music, listening to lines closely related in key and in pitch to previously heard single-line melodies resulted in greater recognition of material among adolescent and adult listeners. Furthermore, in polyphonic listening tasks such as error detection or melody discrimination, higher pitched lines are perceived as more dominant (Fujioka et al., 2005), an effect called “listening up.”

The visual component of conducting is critical for perception of different interpretations; when listeners are given recordings led by different conductors, they cannot discern differences in performance from the audio alone (Madsen et al., 2007, 2009). From the performer’s perspective, visual information is as important as auditory information in ensemble playing. The findings of Fredrickson (1994, p. 314) support this idea that “a combination of aural and visual information can result in better processing of information”.

In Western Art Music, large ensembles typically feature conductors whose gestures are thought to embody structural or expressive elements of the music’s content. As described by conductor Larry Livingston:

…the idea of organizing the music, includes but is not owned or controlled by beating. Because what you’re doing there, you’re trying to work as a congealer of all the different threads that make this special fabric what it is…but in the end, the gestures ought to be the birth children of the music, not the opposite…technique is secondary and primary is to inflect the music. (University of Southern California, 2007)

Though the teaching of conducting often focuses on the movement of the conductors themselves (Green and Gibson, 2004), research is emerging that relates that experience to measurable outcomes, including reactions to gesture among members of the ensemble, evaluators or adjudicators of the ensemble, and the audience. Given the prominent role a conductor plays in large ensemble traditions, it must be asked in what ways and to what degree do conductor gestures influence a musical performance or the perception of that performance by those involved?

As Livingston points out, and conducting pedagogy and practice espouses, conductor gesture serves both a temporal and an inflective or delineative purpose. “A competent conductor must do more than beat time. He or she must interpret the music, reflecting in gesture the style, expression, and dynamics of the score.” (Labuta, 2004, p. 34) Musical gesture can sometimes refer to small moments in the music, expressions of character; an example would be as a “call” and its associated “response.” In conducting, these expressions are reflected in the conductor’s physical movements, or conducting gestures. Gestures may include arm movements, but facial expressions (Wöllner, 2008), eye contact (Price and Winter, 1991), and posture (Van Weelden, 2002) have all also shown to be related to conductor gesture and expressivity. Furthermore, significant effects for other stereotypes, such as race (Vanweelden and McGee, 2007) have been reported, but no significant effects for body type have been documented (Van Weelden, 2002).

Until now, research into conductor gesture congruence and expressivity has largely focused on broad questions of the basic relationship between the presence (or absence) of a conductor’s expressivity and ratings of performance quality, conductor efficacy, and performance expressivity. Further research is needed to investigate the interaction of conductor gesture and listener perception of more specific aspects of music performance.

Much literature exists detailing the relationship between expressive conducting and perceptions of performance. Bender and Hancock (2010) found that a high level of conductor expressivity was rated as more effective than a low level of expressivity, especially when paired with higher quality performances. Further, others have found that degree of expressivity is positively correlated with audience perception of performance expressivity (Morrison et al., 2009; Morrison and Selvey, 2014) as well as with student ensemble members’ evaluations of conductors (Price and Winter, 1991). Conversely, ensemble performance quality may also affect the rating of conductor expressivity (Silvey, 2011). However, Price and Chang (2005) and Price (2006) determined that conductor expressivity does not relate to instrumental ensemble performance adjudication scores, indicating that in certain evaluative contexts there may be more variables at play than conductor expressivity. While much of this literature has used a single “expressivity” rating scale, it is possible that ensemble expressivity ratings are also affected by visual variables or elements outside the performance (such as audience response) on multiple elements of expressivity (Springer and Schlegel, 2016).

Data collected by Meals et al. (2015) indicated that temporal congruence plays a factor in evaluation of the conductor, though not in the manner suggested by some schools of conducting practice. These findings in which both anticipatory and delayed alignment of audio and video were viewed more negatively are contrary to pedagogical practices advocating that conductors should lead the ensemble (Labuta, 2004), thus logically placing gestures slightly ahead of their sonic consequent in time. Nevertheless, the conductor’s role is viewed, in part, as that of guiding an ensemble through a performance and delineating and drawing attention to particular musical moments. Wöllner and Auhagen (2008) found time lags between conductor movements and observer manipulation of a Continuous Response Digital Interface documenting perceptions of expressivity from different perspective points within an ensemble setting. In that study, observers were able to gain specific information about conductors’ expressive musical intentions, even from video-only sequences, and these time lags were largely idiosyncratic reflections of each conductor’s general affective behavior.

Morrison et al. (2014) found that ensemble performances featuring high levels of conductor gestural variability related to specific aspects of performance (articulation and dynamics) were rated significantly more positive than performances featuring low levels of gesture variability for those aspects. These elements of style are content-specific and generally associated with a consistent set of gestural vocabulary, or emblems. Conducting emblems are easily learned and recognized by performers (Sousa, 1988; Mayne, 1992; Cofer, 1998), and it appears that use of such specific gestures may enhance audience members’ experience of a performance, as well. While many previous studies have used participants who were trained in interpretation of conductor gesture (through participation in conducted ensembles; Morrison et al., 2009; Price and Mann, 2011), Price et al. (2016) demonstrated comparable discernment of differences in conductor gesture and effects on ensemble expressivity by non-music majors with no conducted ensemble experience. The use of specific gestural referents raises the question of how the general construct of conductor expressivity might be further understood by examining the congruence between movement and the musical material with which it is associated.

To summarize, previous research has determined that the observation of music performance is a multimodal experience in which both visual and auditory factors play a role. As the embodiment of the music in a large ensemble setting, the conductor is the focal point from which observers may obtain visual information, and thus far research has documented that conductor expressivity does impact performance perception in varied ways. In an effort to further synthesize the specific ways in which conductor gesture may influence perception of a music performance, the purpose of the present study was to determine if differing conductor gesture affected observers’ focus of attention to contrasting aspects of ensemble performances.

Given that expressive gesture plays a role in the perception of performance quality and expressivity, in this study we considered the delineative function of gesture (expressive of specific elements of the score, as suggested in Peddell, 2008 and Price and Mann, 2011), to determine if gestural congruence is a factor in listeners’ perceptions of music content. We predicted that observers’ evaluations of specific elements of a music performance would reflect the aspects of that performance on which conductors focused gestural attention. To compare how changes in congruence to musical material impacts description, we chose two-part excerpts with contrasting features in each line. In these musical situations, conductors choose to present gestural material congruent with some – but not all – material within the musical context, thus comparisons can be made among those differences in gesture and differences in musical material. We hypothesized that as conductors aligned their gesture to one or the other line of the two-part excerpts, participants’ descriptions would align more with features of that musical line.

## Materials and Methods

**FIGURE 1:**
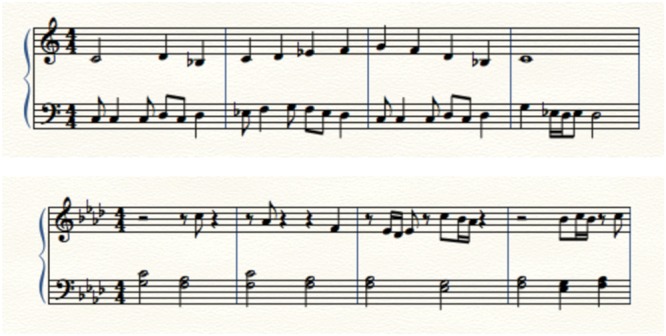
**First four measures of Excerpts 1 (**top**; performed at 120 bpm) and 2 (**bottom**, performed at 60 bpm)**.

We arranged two-part music excerpts for a small conducted ensemble of seven players (flute, clarinet, alto saxophone, trumpet, bass clarinet, F horn, and tuba) convened expressly for this purpose. Excerpt 1 consisted of an ostinato and a lyric melody, and was performed at a tempo of 120 beats per minute. Excerpt 2 included a slow chord progression interspersed with short interjections, and was performed at a tempo of 60 beats per minute. (See **Figure 1** for sample measures of each.) Two arrangements were made of each example, the first with one line assigned to the higher instrument voices and the contrasting line in lower voices, and the second with these assignments flipped, to control for the “listening up” effect (Fujioka et al., 2005). To account for familiarity bias, the excerpts were composed for this experiment and were not based on any specific piece. Experts in the field of wind literature reviewed the excerpts for accuracy, stylistic consistency, and clear contrast between parts; they found the excerpts to be consistent with repertoire of the wind ensemble idiom, and agreed that different gesture content would be appropriate to each line of each excerpt.

Audio was recorded in an empty recital hall using a Zoom 3 audio recorder placed in the seating area approximately 30 ft. beyond the front edge of the stage. Each line of each excerpt was recorded separately and later mixed (using Apple Garageband software) so amplitude of each line could be equalized. The conductors used the *Tempo* (Frozen Ape, 2015) iPhone application’s flashing visual metronome function or audible metronome played over headphones to synchronize tempo in both audio and video recording sessions.

Two conductors were recruited for video-recording of the stimuli excerpts. Conductor one was male, had over ten years experience conducting wind groups, had obtained a master’s degree in wind conducting, and was currently an assistant director of an auditioned wind ensemble on the campus where the recordings were made. Conductor two was female, had over thirty years experience conducting orchestras, had obtained a doctorate in instrumental conducting, and was currently director of orchestras at a high school close to the campus. The researchers knew both to be expressive conductors with great versatility and flexibility to vary their gesture as requested.

We recorded video of each conductor leading the ensemble through all of the excerpt variations, each time altering her or his gesture to emphasize one of the musical lines and directing gestures toward the instruments performing that line. The conductors were instructed to align their gesture to one line or the other in as natural a manner as they deemed appropriate to the score. The conductors varied their gesture by giving visual attention or eye contact to different locations in the ensemble, changes in baton speed, differences in ictus strength, and varied facial expressions. This resulted in 16 distinct videos to be paired with previously recorded audio, two per each conductor, per each excerpt and arrangement, and per each gestural alignment (see **Table 1**).

**Table 1:** Video excerpt recorded conditions and item orders.

	Form 1		Form 2	
	Conductor 1 (F)	Conductor 2 (M)	Conductor 1 (F)	Conductor 2 (M)
Excerpt 1A - Upper voice Ostinato, Lower Voice Melody	(1) Focus on Melody			(6) Focus on Ostinato
	(3) Focus on Ostinato			(8) Focus on Melody
Excerpt 1B – Upper voice Melody, Lower Voice Ostinato		(6) Focus on Ostinato	(1) Focus on Melody	
		(8) Focus on Melody	(3) Focus on Ostinato	
Excerpt 2A – Upper Voice Interjections, Lower Voice Chords		(2) Focus on Interjections	(5) Focus on Chords	
		(4) Focus on Chords	(7) Focus on Interjections	
Excerpt 2B – Upper Voice Chords, Lower Voice Interjections	(5) Focus on Chords			(2) Focus on Interjections
	(7) Focus on Interjections			(4) Focus on Chords

Recordings were made using a Sony HD Handycam video camera. To capture as much visual information as possible, we recorded the conductors from the perspective of the ensemble rather than the perspective of the audience; the camera was placed behind and to slightly off-center of the two rows of musicians, and remained in a stationary fixed position (no zoom or camera panning.) Although the performer’s perspective is not a native viewpoint for most audiences, all participants in the present study were experienced ensemble performers and were familiar with this viewing perspective. Its use here was consistent with other research utilizing observer perspectives of conductors (Morrison et al., 2009, 2014). Previous findings have concluded that although conductor evaluations differ in magnitude (but not valence) by viewing angle (Peddell, 2008; Price and Mann, 2011), the performer’s perspective is richer with visual stimuli which are necessary for such finely discriminating tasks as examining differences in gesture congruence.

Video excerpts were then digitally edited to overlay video with the mixed audio using Apple Final Cut Pro 10.2. Audio remained the same across conductors and conducting conditions, with minor adjustments (trimming of silences/spaces within the audio) to account for temporal fluctuation. Total run time for videos of Excerpt 1 ranged from 34 to 37 s, and for Excerpt 2 ranged from 67 to 93 s. Although audio adjustments did result in a few sonic artifacts, participants of a pilot test of the instrument did not indicate those artifacts to be noticeable.

Final versions of the 16 videos were embedded in a web survey (Learning and Scholarly Technologies, 1998) using YouTube. The video items were ordered in two test forms with the stipulations that participants did not see the same conductor twice in a row. To avoid confounding gesture difference and conductor, each test form included each conductor in both conductor conditions (focus of gesture on one or the other musical line) for only one of the excerpt voicings (see **Table 1**). A sample video featuring a different conductor in a similar conducting venue and position was used to familiarize participants with the format of the survey. Three independent researchers were recruited to pilot-test the instrument and provide feedback; all indicated that the instrument was easy to use and that the video stimuli were effectively presented.

After each video, participants were prompted with four 10-point bipolar rating scales for the elements of Articulation (*disconnected* to *connected*), Rhythm (*irregular* to *regular*), Style (*angular* to *flowing*), and Phrasing (*short* to *long*.) These items were similar to the bipolar scales used by Repp (1990) and Bergee (1995) in previous studies of music performance evaluation. These four elements were chosen based on two criteria: Firstly, they are all categories that frequently appear on ensemble adjudication forms in some way or have been used in research into ensemble evaluation (Bergee, 1995; Springer and Schlegel, 2016); and secondly, each are elements considered to be controllable by conductor gesture (Green and Gibson, 2004).

**FIGURE 2:**
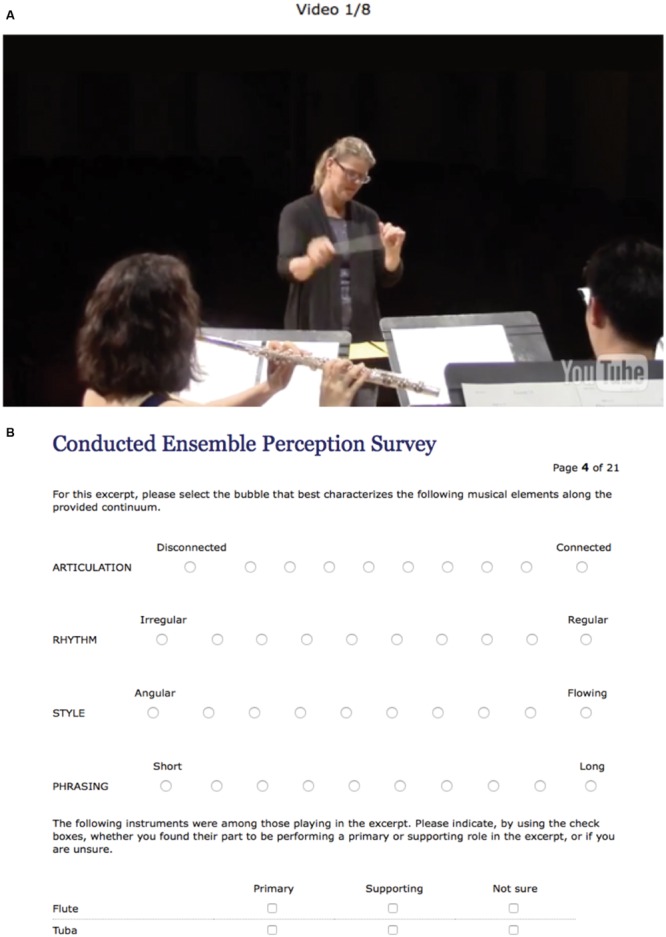
**(A)** Sample of video stimulus in response instrument. **(B)** Sample of items in response instrument (Catalyst Web survey).

Participants were also asked to characterize the line played by the flute and the line played by the tuba as either “primary” or “supporting”, or indicate that they were unsure. This was to determine if conductor gestural attention toward the upper or the lower instruments (playing one of the two excerpt lines) had any relationship to the descriptive ratings. The domain terms and dichotomous anchors were not defined for participants; when questions arose, participants were directed to use their best judgment and rely on their own interpretation of the term. See **Figures 2A,B** for a sample of the response instrument.

Participants (*N* = 42) were recruited from instrumental ensembles at a large university and a community ensemble known to the researchers in the Pacific Northwest region of the United States. They ranged in age from 18 to over age 50, and had between 3 and 17 years experience performing in a variety of conducted ensembles (concert band, marching band, orchestra, choir, jazz ensemble, and other unspecified ensembles; *M* = 7.81, *SD* = 4.02). Most did not have any conducting experience, but a few *(n* = 4) were former music educators with conducting coursework and experience. Participants were recruited specifically from ensembles in which the concepts of the expressive rating scales, *articulation, rhythm, style*, and *phrasing* would be frequently presented in the rehearsal setting, and therefore adequately understood in context of viewing a conductor.

All procedures were approved and carried out according to our university’s Institutional Review Board protocols. Participants were randomly assigned to one of the two survey forms. Surveys were completed in either a university computer lab or on laptops in the offices of the community music school where the community ensemble rehearses. To minimize interference with other participants, all were provided headphones to complete the task. On average, participants took about fifteen minutes to complete the entire survey instrument.

## Results

Data consisted of ratings of each excerpt for each of the four variable scales (Articulation, Rhythm, Style, Phrasing). Ratings were converted to numbers from 1 (*disconnected, irregular, angular, short*) to 10 (*connected, regular, flowing, long*). We predicted that ratings would differ depending on conductor gesture and would reflect the musical line toward which the conductor focused gestural attention. Independent samples *t-*tests of between-form contrasts revealed no significant differences based on test form for each of the 10-point descriptive scales (*p* > 0.05 for all contrasts). We therefore used a mixed linear model to determine the degree to which gesture, conductor, and excerpt arrangement, as well as interactions between gesture/conductor and gesture/excerpt arrangement, were factors in ratings for each of the four dichotomous rating scales. Mixed models allow the use of both fixed and random independent variables in the same model, and also allow for nesting (incomplete, or not fully crossed) as in this case with participants nested within separate test forms. This model also accounted for individual differences between participants by treating each item rating as an individual case, thus allowing for interpretation of the ratings independent of the random variable of participants (each of whom may have had a different skew toward his/her rating).

**FIGURE 3:**
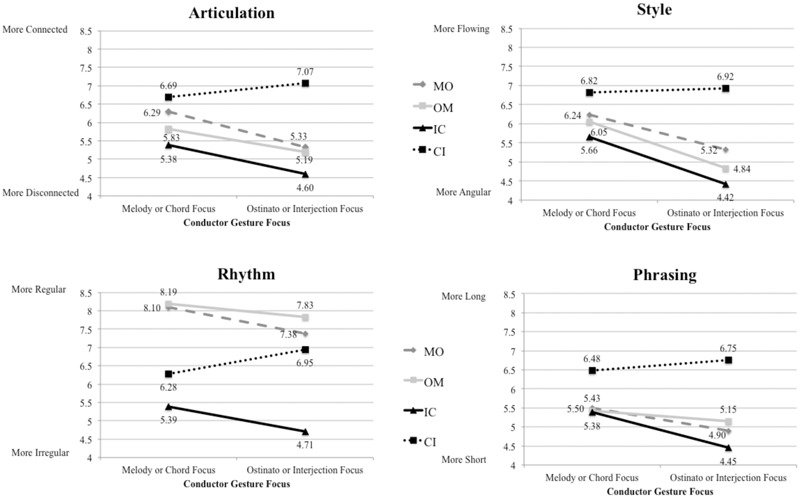
**Mean ratings by gesture focus along each dichotomous scale for each arrangement of excerpts: MO = Excerpt 1, Melody upper voices, Ostinato lower; OM = Excerpt 1, Ostinato upper, Melody lower; IC = Excerpt 2, Interjections upper voices, Chords lower voices; CI = Excerpt 2, Chords upper, Interjections lower**.

Comparisons of average ratings for each of the four dichotomous variable scales are presented in **Figure 3**. Overall, we observed differences in evaluations of Excerpt 1 (lyric melody and ostinato) according to expectation. When conductor gesture was congruent with the ostinato, participants rated the excerpt as having more *disconnected* Articulations, *irregular* Rhythm, *angular* Style, and *short* Phrases. When gesture was congruent with the melody, ratings indicated more *connected* Articulation, *regular* Rhythm, *flowing* Style, and *long* Phrasing. This result was evident regardless of arrangement (with the melody performed by either the upper voices or lower voices, labeled “MO” and “OM”, respectively, in **Figure 3**). However, these differences were only significant for articulation [*t*(285) = 2.53, *p* = 0.012] and style [*t*(285) = 3.50, *p* = 0.001] rating scales, and not for the rhythm or phrasing scales (*p*-values > 0.05). Ratings of Excerpt 2 (chords with interjections, labeled “CI” and “IC”) showed a less consistent relationship with gesture, with the arrangement featuring upper voice chords and lower voice interjections (“CI”) evoking evaluations contrary to expectation. None of these differences based on gesture were significantly different (*p* > 0.05).

Across all excerpts, main effects were reported within each rating scale. For the *articulation* scale, significant main effects were found for Conductor [*F*(1,285) = 5.99, *p* = 0.015], Gesture Direction [*F*(1,285) = 3.29, *p* = 0.039], and Arrangement [*F*(1,285) = 11.29, *p* = 0.001] and there was an expected significant interaction between Arrangement and Excerpt (*F*(1,285) = 22.13, *p* < 0.001). No significant interaction was found between Conductor and Gesture (*p* = 0.58) indicating that differences in gestures between conductors were not attributable to differences in Conductors themselves. For the *rhythm* scale, significant main effects were found for Conductor [*F*(1,285) = 13.54, *p* < 0.001] and Arrangement [*F*(1,285) = 13.14, *p* < 0.001], and a significant interaction was found between Arrangement and Excerpt [*F*(1,285) = 5.58, *p* = 0.019]. No significant main effect was found for Gesture (*p* = 0.148) nor for the interaction term of Gesture by Conductor (*p* = 0.823). For the *style* scale, significant main effects were again found for Arrangement [*F*(1,281.32) = 12.77, *p* < 0.001] and Conductor [*F*(1,281.35) = 9.61, *p* = 0.002], and a significant interaction was found between Arrangement and Excerpt [*F*(1,281.32) = 23.11, *p* < 0.001]. The *style* scale also featured a significant effect for Gesture [*F*(1,281.32) = 5.52, *p* = 0.004], but again, not for the Gesture by Conductor interaction (*p* = 0.186). Finally, for the *phrasing* scale, a significant main effect was found for Arrangement [*F*(1,282.89) = 14.54, *p* < 0.001] and a significant interaction was found for Excerpt by Arrangement [*F*(1,282.21) = 14.25, *p* < 0.001] However, no main effects were found for Conductor (*p* = 0.136), Gesture (*p* = 0.139) or the interaction between the two (*p* = 0.281).

Model estimates (effects) between excerpts, arrangements, and conductors are reported in **Table 2**. The model confirmed a significant difference between excerpts. There was also a significant effect for voicing arrangement, with both the “MO” arrangement (in which the melody was in the upper voices and the ostinato in the lower) and the “IC” arrangement (in which the interjections were in the upper voices and chords were in the lower voices) differing from their contrasting arrangements, regardless of gesture condition. We observed a significant interaction between excerpt and voicing arrangement reflecting differences between the more consistent “MO/OM” response patterns and the contrasting “CI/IC” patterns. Although there was a significant difference between conductors on the domains of Rhythm and Style, this difference did not interact with gesture (see **Tables 3** and **4**).

**Table 2:** Model estimates (β): mean rating differences for excerpts, arrangements, and conductors (Confidence Interval in parentheses).

Rating Scale	Excerpt 1 over 2	Arrangement A over B	Interaction Excerpt × Arrangement	Conductor Female over Male
Articulation	-2.14^∗∗^ (-2.44, -0.47)	-1.79^∗∗^ (-2.48, -1.25)	2.08^∗∗^ (1.26, 3.01)	1.05^∗^ (0.17, 1.92)
Rhythm	1.32^∗^ (0.37, 2.28)	-1.30^∗∗^ (-1.90, -0.69)	1.02^∗^ (0.17, 1.88)	0.60 (-0.26, 1.45)
Style	-1.45^∗^ (-2.44, -0.47)	-1.86^∗∗^ (-2.48, -1.25)	2.13^∗∗^ (1.26, 3.01)	1.05^∗^ (0.17, 1.92)
Phrasing	-1.17^∗^ (-2.17, -0.19)	-1.70^∗∗^ (-2.33, -1.08)	1.69^∗∗^ (0.81, 2.58)	0.36 (-0.52, 1.24)

*^∗^*p* < 0.05; ^∗∗^*p* < 0.001.*

**Table 3:** Model estimates of factors within Excerpt 1 “MO/OM” (β): Gesture and Gesture/Conductor Interactions (Confidence Interval in parentheses).

Rating Scale	Gesture Melody over Ostinato	Interaction Gesture × Conductor
Articulation	1.12^∗^ (-0.59, 1.12)	-0.64 (-0.66, 1.75)
Rhythm	0.26 (-0.59, 1.12)	0.55 (-0.66, 1.75)
Style	1.55^∗^ (0.26, 2.42)	-0.45 (-2.36, 0.12)
Phrasing	0.88 (-0.01, 1.76)	-0.59 (-1.84, 0.66)

*^∗^*p* < 0.05; ^∗∗^*p* < 0.001.*

**Table 4:** Model estimates of factors within Excerpt 2 “CI/IC” (β): Gesture and Gesture/Conductor Interactions (Confidence Interval in parentheses).

Rating Scale	Gesture Chords over Interjections	Interaction gesture × conductor
Articulation	-0.50 (-2.01, -0.11)	-0.55 (-1.06, 1.35)
Rhythm	0.26 (-0.59, 1.12)	-0.14 (-1.06, 1.35)
Style	0.64 (-0.24, 1.52)	-0.45 (-1.69, 0.78)
Phrasing	0.65 (-0.24, 1.53)	-0.15 (-1.39, 1.10)

*^∗^*p* < 0.05; ^∗∗^*p* < 0.001.*

Differences in gesture within excerpts are presented in **Tables 3** and **4**. The only significant differences were in the domains of Articulation and Style in Excerpt 1 (melody/ostinato). Gesture clearly played a delineative role in these domains. There were moderate positive correlations between Articulation and Style scores (*r* = 0.60, *p* < 0.01), and Style and Phrasing scores (*r* = 0.51, *p* < 0.01). There were no significant differences for gesture in Excerpt 2 (chords/interjections) in any of the four domains. This result is not surprising due to the contrasting response patterns between the two voicings of this excerpt (**Figure 3**). No significant effects were observed for the domains of Rhythm (*p* = 0.546 for both excerpts) and Phrasing (*p* = 0.053 for Excerpt 1, and *p* = 0.15 for Excerpt 2) for either excerpt, though responses in these domains generally resembled those observed for Articulation and Style.

**FIGURE 4:**
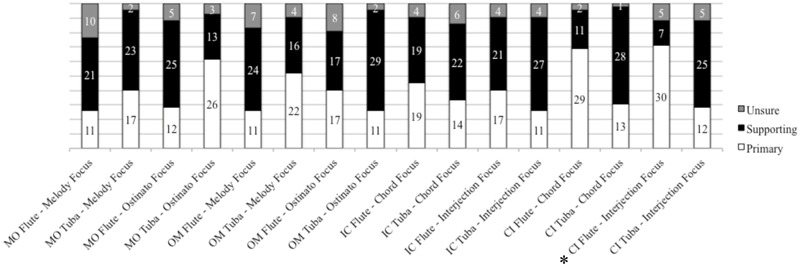
**Frequencies of instrument dominance ratings (*^∗^p* < 0.003)**.

Instances of categorization of the flute voice or tuba voice as “primary”, “supporting”, or “unsure” are presented in **Figure 4**. Overall, the timbre dominance questions yielded little relationship with gesture focus. We used Chi-square goodness-of-fit tests to determine cases in which selections of “primary” and “supporting” were different than chance. Using a Bonferroni correction for multiple comparisons (adjusted α = 0.003), only participants’ identification of flute as the primary voice in the “CI” excerpt for which conductors focused on the lower “interjections” line was significant [χ^2^(1) = 14.30, *p* = 0.003]. Overall for this excerpt, the tendency was for participants to rate the upper voice as primary regardless of gestural focus of attention. This may be indicative of a bias toward upper voiced instruments in this excerpt and may explain the relative lack of variability among the descriptive ratings for this voicing regardless of accompanying visual information. We speculated that the focus of the gesture would be associated with an increase in primacy ratings for the aligned voice. However, this was rarely the case. While there was a trend toward such a ratings profile for Excerpt 2 “IC”, with gesture aligned to the interjections in the flute voice, the majority of rankings still identified both timbres as secondary. In some cases, ratings were opposite to gesture indications. For example, in Excerpt 1 “MO”, with the melody in the upper voice and gesture aligned to that part, participants did not rank the flute voice as primary. In the same voicing condition (flute with melody) but different gesture condition (gesture aligned to the ostinato/lower voice) the majority of participants did identify the tuba voice as primary. In some cases, primary/secondary rankings were equivalent, indicating that overall participants found neither voice to be more pronounced, regardless of gesture.

## Discussion

Conducted ensembles offer an interesting avenue for the investigation of the manner in which visual and auditory information interact in the context of music performance. The conductor, in particular, is somewhat unique among performing musicians in that her/his role in a music performance is entirely visual in nature, from the perspective of the audience. The purpose of this study was to determine if observers’ perceptions of musical content were related to conductor gesture. Previous research has documented the powerful role of visual information in the evaluation of music performances (Tsay, 2013). Most of the findings related to this topic have examined the broad construct of expressive movement and have compared performances either with or without accompanying expressive gesture (e.g., Morrison et al., 2009), with varied levels of expressive gesture (e.g., Vines et al., 2011), or with varied levels of performance quality (e.g., Silvey, 2011). Here, rather than examine the presence, absence or relative magnitude of expressive gesture, we considered whether the content of musical gesture was related to the way in which one understood a music performance. We predicted that a conductor’s gestural focus on particular aspects of a piece of music would affect the way an observer described the performance. These effects are evident because here, as in other previous studies (Morrison et al., 2009, 2014; Price and Mann, 2011; Price et al., 2016) the variation in the visual component of stimuli (video recorded conductor gesture) was what elicited the response effect, whereas the audio within the stimuli remained constant.

The present data suggest that conductor gesture does appear to have an effect on observer perception. Consistent with previous research, the visual aspect of conducting plays a consequential role in the perception of a conducted ensemble’s performance (Madsen et al., 2007, 2009; Price and Mann, 2011; Price et al., 2016). The relationship between conductor gesture and observer description was particularly evident in the domains of articulation and style, aspects of music performance that have been reported elsewhere to yield notably different responses from listeners depending on the movement accompanying them (Morrison et al., 2014). Rather than being consistent in its impact, however, this effect appears to be mediated by preconceptions of the importance of melodic over rhythmic material, of certain timbres over others, and of the durations between onsets of new active material.

For the first of the music excerpts we examined in which there was a lyrical melody and rhythmic ostinato, our findings largely conformed to expectations. When gesture was aligned to the melody, evaluations tended more toward *connected, regular*, and *flowing*, and indicated *long* phrases. Conversely, when gesture was aligned with the ostinato, participants tended to describe the music as *disconnected, irregular*, and *angular*, with *short* phrases. Although the content of the performances did not change, the way in which participants described the music tended to reflect the aspects of the music to which the conductor gave gestural attention. It may be that the conductor’s movements served to delineate and figuratively amplify one musical structure over another. Given the array of musical information vying for an audience member’s attention, the conductor may serve as a guide to that which is most salient at the moment. The congruence of the conductor’s expressive movement (for example, smooth and flowing) with certain features of the musical landscape (a lyrical melody) may reinforce certain movement-based imagery evoked by the corresponding set of sounds (Eitan and Granot, 2006) and propel this material to the forefront of the audience member’s attention. While this study utilized a viewing perspective not usually available to audiences, in light of these findings and those of Peddell (2008), a case could be made for audiences to be afforded the opportunity to view conductors from a performer’s perspective more often, in order to take advantage of the rich variety of visual information available. Conversely, utilizing the present study design from the traditional viewing angle of an audience member may serve as a way to isolate gestures emanating from the arms and torso from more performer-directed visual information such as facial expression and eye contact.

In contrast, in our second excerpt in which there were flowing chords overlaid with seemingly random interjections, participants’ descriptions did not consistently follow the conductor’s lead. When the rhythmic interjections appeared in the top voice and were reinforced by the conductor’s gesture, listeners described the music as *disconnected, irregular*, and *angular*, with *short* phrases. This was not the case when the same material appeared in the lower voice even when supported by the conductor’s movements; descriptions were, instead, consistent with the musical material appearing in the upper voices. In this particular juxtaposition of material, listeners tended to keep attention fixed on the upper voice, as reflected in their identification of the flute voice as primary across both arrangements and gesture conditions. Unlike in the first excerpt, here the conductor gestures may not have been either as clear or as helpful. In terms of clarity, the isolated character of the rhythmic interjections may have been viewed as somewhat at odds with the necessarily continuous movement of the conductor, thus potentially weakening the link between visual and auditory information. Alternatively, the distinction between the more connected chordal line and the more detached rhythmic interjections may have been clear enough that the listeners could easily identify and track the voice that they felt was the better candidate for the primary role, a case of easily being able to “listen up” (Fujioka et al., 2005).

These discrepant findings between the two excerpts suggest that, while conductor gesture does impact perceptions of performance, there are a number of variables that observers may perceive and use to understand and organize the music they hear. Visual components of music performance can effectively transmit aspects of structure and form (Thompson et al., 2005; Connell et al., 2013) but it may be that conductor gesture is more delineative for music with shorter metric periods and more melodic material; the longer periodicity and slower tempo of our second excerpt may have limited participants’ ability to react to visual differences (Luck et al., 2010) or may have proven difficult to organize due to the less structured character of the more rhythmic line (such as described by Gritten, 2011). Conductor gesture aligned to the chordal line of Excerpt 2 was much slower and had fewer immediate changes or musical events with which to construct meaning, compared to the faster and more visually active Excerpt 1. Musical material that offers frequent repeated opportunities for gestural depiction (perhaps such as the articulation and stylistic parameters of the first excerpt) may more readily accommodate the integration of auditory and visual information, much like in cases where changes within a music parameter evoke strong visual associations (Eitan and Granot, 2006) or are clearly detected when supported by gesture (Morrison et al., 2014).

Responses relating to articulation and style were moderately positively correlated, raising the question of whether respondents viewed these elements of gesture, or of performance, similarly. Though the descriptive anchors for each scale were different (*disconnected* to *connected* versus *angular* to *flowing)*, the gestural enactment of these elements is typically similar (in conducting pedagogy, described as coming from the baton’s resistance and speed; Labuta, 2004). Participants were not provided definitions for any of the terms used in the response items, but all had ensemble performance experience and therefore likely had familiarity with the concepts. The similarity between these ratings, and lack of significant difference in the Phrasing and Rhythm scales, may indicate that participants focused less on aspects of gesture related to these concepts and more to their perceptions (and preconceptions) of the music. While the same might be said for untrained observers of conductors, this study used participants familiar with conductor gesture in a responsive way, as current members of conducted ensembles themselves, so as to elucidate a more naturalistic reaction to conductor gestures. However, untrained observers may have similar reactions to differences in conductor gesture, given explanation of the rated elements, as non-music majors have demonstrated similar sensitivity to levels of conductor expressivity in rating both conductor and ensemble expressivity (Price et al., 2016).

The conductors were instructed to use gestures aligned to one or the other line of each excerpt as they saw fit. The degree of difference between them was consistent with what might be expected between any two expert musicians, and it is unlikely that the difference in participants’ responses by conductor was not attributable to any specific hand or arm movements, points of eye contact, or facial expressions. This lack of specificity was by design, but may also be why the degree of difference between gesture conditions was relatively minor for many of the comparisons. Previous research (Morrison et al., 2009, 2014; Bender and Hancock, 2010) has found that more expressive conductors are rated as more effective, but has not examined how conductor gestural choices, taken from a number of viable options, may be perceived in relation to effectiveness. Further research is needed to explore the way in which descriptive ratings may interact with evaluations of conductor efficacy.

Price and Mann (2011, p. 69) determined, “it is clear that conductors have an effect on the way a performance sounds to an audience”. They cautioned that empirical evidence did not support the conclusion that conductors have a systematic effect on a performance itself, but are rather one component of an experience that individuals construct through multiple modalities. Our findings support this claim and further clarify the ways in which gesture may impact one’s perception of a performance. Much as in the case of solo performers (Davidson, 2012), it is conceivable that, in working with an ensemble, a conductor might employ differences in gesture to highlight specific musical material within the context of a polyphonic work. Our data suggest that, in certain contexts, this visual information may shape the way audience members or other members of the performing ensemble hear, recognize, or organize complex music. These results add to the body of literature that supports an interpretation of the visual domain as critical to the perception of music (Platz and Kopiez, 2012). In the context of the large ensemble, conductor gesture plays its part through the broad lens of expressivity, and on a more fine-grained scale through its interpretive or delineative qualities.

## Author Contributions

Both authors AK and SM certify that they share full authorship of this manuscript. Both contributed substantially to the conception and design of the study, drafting and revising of this manuscript, will be responsible for final approval, and agree to be accountable for all aspects of the work.

## Conflict of Interest Statement

The authors declare that the research was conducted in the absence of any commercial or financial relationships that could be construed as a potential conflict of interest.
